
JOURNAL INFORMATION
==============================
NLM Title Abbreviation: ScientificWorldJournal
Journal ID: ScientificWorldJournal
NLM Journal ID: 101131163
Journal ID: TSWJ

The Scientific World Journal

ISSN: 2356-6140
EISSN: 1537-744X
Publisher: Wiley

ARTICLE INFORMATION
==============================
PMCID: PMC6009226
PMID: 29973824
DOI: 10.1100/tsw.2002.113
Article ID: 316757
Article version: 1
Subjects: Editorial

Linking Science with Management of Freshwater Resources – A Forthcoming Collection of Papers in the Freshwater Systems Domain

Havens Karl E. *
South Florida Water Management District, West Palm Beach, FL 33406, USA
*Karl E. Havens: khavens@ifas.ufl.edu
Publication date: 2002
Electronic publication date: 2002 Jan 12
Volume: 2
First page: 105
Last page: 106
Received 2002 Jan 2; Accepted 2002 Jan 2
Copyright: Copyright © 2002 Karl E. Havens.
Copyright year: 2002
License: This is an open access article distributed under the Creative Commons Attribution License, which permits unrestricted use, distribution, and reproduction in any medium, provided the original work is properly cited.
License URL: https://creativecommons.org/licenses/by/3.0/

Keywords: water resources, environmental management, aquatic sciences, resource management, ecosystem restoration, reservoir water quality

==============================
Research Article
TheScientificWorldJOURNAL (2002) 2, 105-106
ISSN 1537-744X; DOI 10.1100/tsw.2002.113

### Linking Science with Management of Freshwater Resources – A Forthcoming Collection of Papers in the *Freshwater Systems* Domain

Karl E. Havens
*South Florida Water Management District, West Palm Beach, FL 33406*

**E-mail:** khavens@ifas.ufl.edu

*Received January 2, 2002; Accepted January 2, 2002; Published January 12, 2002*

**KEY WORDS:** water resources, environmental management, aquatic sciences, resource management, ecosystem restoration, reservoir water quality

**DOMAINS:** freshwater systems, ecosystems and communities, environmental management and policy

With a rapidly growing human population and demand for freshwater, the world's lakes, rivers, and wetland ecosystems are being impacted at an ever-increasing rate. At no time in the past has there been a greater need for sound management of these aquatic resources and their watersheds, and for integration of science with resource management. Yet at a time when there is a dire need for water management agencies and scientific institutions to invest in more research, especially in water-stressed areas, just the opposite may be occurring. Budget cuts, changes in government policy, and other factors risk reduction, rather than strengthening, of the role of hypothesis-driven research in ecosystem management.

A special session at the 2002 meeting of the American Society of Limnology and Oceanography (ASLO, June 9–14, Victoria, B.C.) will address this important issue. The session, titled "Linking Science with Management of Freshwater Resources," supports the over-arching theme of the ASLO meeting — inter-disciplinary linkages in the aquatic sciences and beyond. The session will include a keynote address by a leading expert in science-management linkages at the global scale, followed by several focused study papers that address specific cases where hypothesis-driven research is facilitating the effective management of freshwater systems. Topics will include development of reservoir water quality management plans based on empirical models for taste and odor, use of state/national partnerships in ecosystem restoration science, and use of ecosystem models for establishing hydrologic restoration goals in large lakes. Additional details regarding the 2002 ASLO meeting can be found at the conference web site: www.aslo.org/victoria2002

Research papers from this session will be peer-review and published in the *Freshwater Systems* domain of TheScientificWorld.

Karl E. Havens Principal Editor, *Freshwater Systems* January 2002 METHODS

Email: khavens@ifas.ufl.edu.
©2002 with author.

**This article should be referenced as follows:**

Havens, K. (2002) Linking science with management of freshwater resources — a forthcoming collection of papers in the *Freshwater Systems* domain. *TheScientificWorldJOURNAL* **2**, 105–106.
